# The impact of management practices on relative patient mortality: Evidence from public hospitals

**DOI:** 10.1177/09514848211068627

**Published:** 2022-02-17

**Authors:** Reza Salehnejad, Manhal Ali, Nathan C Proudlove

**Affiliations:** 1 66058 University of Manchester Alliance Manchester Business School, Manchester, UK; 2 4468 University of Leeds, Leeds, UK; 3 5292 University of Manchester, Manchester, UK

**Keywords:** hospital performance, quality, mortality, management practices, machine learning, panel data analysis

## Abstract

A small, but growing, body of empirical evidence shows that the material and persistent variation in many aspects of the performance of healthcare organisations can be related to variation in their management practices. This study uses public data on hospital patient mortality outcomes, the Summary Hospital-level Mortality Indicator (SHMI) to extend this programme of research. We assemble a five-year dataset combining SHMI with potential confounding variables for all English NHS non-specialist acute hospital trusts. The large number of providers working within a common system provides a powerful environment for such investigations. We find considerable variation in SHMI between trusts and a high degree of persistence of high- or low performance. This variation is associated with a composite metric for management practices based on the NHS National Staff Survey. We then use a machine learning technique to suggest potential clusters of individual management practices related to patient mortality performance and test some of these using traditional multivariate regression. The results support the hypothesis that such clusters do matter for patient mortality, and so we conclude that any systematic effort at improving patient mortality should consider adopting an optimal cluster of management practices.

## Introduction

Significant variations in hospital performance are pervasive and recognised as a major concern.^1–3^ In England, hospitals are managed by trusts, each with one or more hospital sites. In this paper, we examine the Summary Hospital-level Mortality Indicator (SHMI), the standard metric of relative mortality across NHS hospital trusts in England, over a period of 5 years. The data exhibit considerable variation, which is persistent over time, and offer opportunities for improving health outcomes by understanding why some trusts consistently do better or worse than others.^4^ This is the focus of this paper.

The empirical literature points to a multitude of factors that influence patient safety and mortality across hospitals. They include the role of incident reporting,^5,6^ patient education,^7^ staff satisfaction^8^ and workload,^9^ culture^10^ and hospital board quality.^11^ Despite some progress, issues surrounding drivers of patient safety and mortality are not settled. Neither is the relationship between safety metrics and mortality: Howell et al.^5^ found no association between safety and mortality outcomes in a sample of data on NHS trusts; Mannion et al.^11^ assess board competency in NHS hospital trusts and find strong association with safety culture, but not with metrics such as mortality and infection rates.

The recent literature on hospital performance points to the importance of management practices in driving health outcomes^12–21^: effective management and organisation of resources drive higher-quality patient outcomes, including lower risk-adjusted hospital mortality from acute myocardial infraction.^22–24^ The literature, though, has looked into the role of management practices as a composite or as individual practices separately.^25^ Relatively little attention has been paid to *interactions* among management practices in driving outcomes such as relative mortality. Management practices are likely to have a higher impact on performance when they are implemented in particular configurations.^26^

This paper explores impacts of alignments of management practices on patient mortality. Our conjecture is:

*Research Hypothesis*: Alignments of management practices matter in driving relative excess mortality, with some leading to outcomes materially better than others.

We examine this hypothesis using a dataset on English NHS trusts. In the United Kingdom, the NHS provides the very great majority of healthcare, free at point of use, funded by general taxation. For most cases, the patient’s first contact is via primary care, who may send them to a hospital (secondary-level care) for urgent care or refer them to a hospital specialist for consideration for planned care. Hospitals are managed by acute hospital trusts, each running one or more hospital site. We collect data on the SHMI, management practices and relevant control variables covering 5 years – a longitudinal or ‘panel’ dataset. We employ an unbiased regression tree technique to identify alignments among the management variables and test their significance for relative mortality. Our research contributes to a growing literature that associates variations in hospital performance with differences in management practices.^23,27–30^ The literature contains increasing evidence of the impact of management practices^22,28,29^ together with some natural^31^ and designed randomised controlled experiments^32^ to investigate causality.

## Methods

### Data & descriptive statistics

Avoidance of unnecessary deaths is a prominent quality metric for acute healthcare providers. To measure relative hospital mortality, we use the Summary Hospital-level Mortality Indicator (SHMI), ‘calculated as the ratio between the actual number of patients who die following hospitalisation at the trust and the number that would be expected to die on the basis of average England figures, given the characteristics of the patients treated there’.^33^ Expected deaths is estimated using all inpatient deaths up to 30 days after discharge with risk adjustment for case-mix factors such as patient age, sex, ethnicity, deprivation, admission type and history and co-morbidity to provide fairer comparisons across hospitals.^33^ The SHMI has been used widely in academic research as a measure of hospital mortality.^25,34–37^

**Figure 1.**
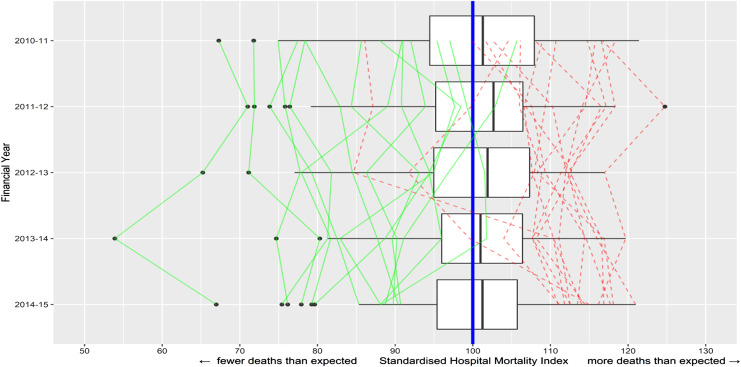
Relative mortality (SHMI) over the period 2010/11 to 2014/15 for the 133 NHS acute hospital trusts with data for all 5 years. The vertical blue line is the expected level (SHMI = 100) (note the black lines in the boxes are the medians for each year). The boxplots show considerable variation. Green/solid lines connect the historical path of each of the trusts in the best decile *in 2014–2015*; red/dashed of each in the worst decile.

We use SHMI for the years 2010/11 to 2014/15. For convenience, we multiply the SHMI by 100, so that the numbers can be interpreted as percentages of the baseline (i.e. of deaths at the expected level). Figure 1 uses boxplots to show the wide dispersion in SHMI across hospital trusts in each financial year of our dataset. The ratio between the 90*th* and 10*th* percentiles of the SHMI distribution in the sample is 1.275 (1.12/0.877), suggesting that the patient mortality is around 25*%* lower in better performing trusts. The graph also adds line plots to connect the SHMI year-by-year for those trusts that ended up being in the best or worst 10*%* of all trusts in the final year (2014/15) – that is, it traces back their trajectories, showing considerable performance persistence over time. A statistical measure of this persistence is the autoregression coefficient from regressing the SHMI metric against its 1-year lag. This yields a coefficient 0.848, significant at 1*%* (perfect correlation from year to year would give a coefficient of (1). Thus, a high- or low-performing hospital trust 1 year is very likely to remain high- or low performing in subsequent years. Similar relative-performance persistence across NHS hospitals has been found in, for example, elective operation cancellation rates.^38^

Our management practices variables are derived from the annual NHS Staff Surveys (NSS), which all NHS trusts are required to administer. NSS are available back to 2003, weighted according to trust size and staff composition to increase comparability.^39^ We collect data for the years 2009/10 to 2013/14. Across these years, the total numbers of respondents were from 116, 000 to 250, 000, representing mean overall response rates of between 49*%* and 52*%*. Missing responses on the individual variables of interest to us range from around 12*%* to under 1*%*, so missing data is a fairly small proportion of large response volumes. The available data are the averages of responses for each trust.

NSS captures views of clinical and non-clinical staff on a range of issues related to work experiences and well-being. It includes questions relating to human resource management culture which allow comparisons between trusts. Of principal interest to us are four variables: the decentralisation of decision making (*Decisions*), effective communication between senior management and staff (*Communication*) and whether senior managers act on staff feedback (*Feedback*) and on suggested ideas for improving services (*Ideas*). These four practices constitute some of the key elements of Appelbaum et al.’s high-performance work system^40^ and measure staff perceptions of the management environment. Responses on these NSS questions are Likert-type variables measured on a five-point scale from strongly disagree to strongly agree. We construct scores for these practices by taking their positive response rate (PRR), that is, the percentage of respondents who agree or strongly agree, as used by other researchers.^8^

Our analysis also includes the following additional variables from the NSS: *Flexible* working practices, *Workplace pressure*, *Incident* reporting, *Job Design*, *Appraisal*, *Supervisor* and *Team Quality*. Table 1 defines these variables. Job design is particularly interesting: high quality on this aspect is associated with healthcare staff engagement and satisfaction, which, in turn, can affect their own health and on the quality of care delivered to the patients.^41^ The current *Job Design* variable in NSS is a composite of six different measures (as shown in the table), each on a scale 1–5, that capture quality and clarity of job content.

**Table 1.** Data definitions and sources.

*Data*	Definition	Source
***Dependent variable***		
SHMI	Summary hospital mortality index × 100	NHS Digital
***Trust structure***		
Teaching	1 if trust is teaching trust, otherwise 0	www.nrls.npsa.nhs.uk
Foundation trust	1 if trust has FT status, otherwise 0	Monitor
Beds	Average number of available beds	NHS England
Daycase rate	Proportion of activity carried out as daycases. General and acute elective admissions: Daycases/total	Dept. of health monthly hospital activity data
***Workforce skill-mix***		
Medical staff (%)	(Medical workforce /total staff) × 100	NHS workforce statistics
Nursing staff (%)	(Nursing workforce /total staff) × 100	NHS workforce statistics
Support staff (%)	(Support staff /total staff) × 100	NHS workforce statistics
***Management & organisational***		
Management: Decisions	‘Senior managers here try to involve staff in important decisions’ (PRR)	NHS staff surveys (NSS)
Management: Feedback	‘Senior managers act on staff feedback’ (PRR)	NSS
Management: Ideas	‘Senior managers encourage staff to suggest new ideas for improving services’ (PRR)^a^	NSS
Management: Communication	‘Communication between senior management and staff is effective’ (PRR)	NSS
Flexible working practices	% using flexible working options^a^	NSS
Job design	Quality of job design (weighted average of: I have clear, planned goals and objectives for my job; I often have trouble working out whether I am doing well or poorly in this job; I am involved in deciding on changes introduced that affect my work team; I always know what my work responsibilities are; I am consulted about changes that affect my work area; and I get clear feedback about how well I am doing my job – each score 1–5; 5 is the highest quality)^a^	NSS
Team quality	Effective team working (summary score 0–100)^a^	NSS
Appraisal	% having well-structured appraisal reviews within previous 12 months	NSS
Supervisor	Support from immediate managers (score 1–5)	NSS
Incident	Fairness and effectiveness of procedures for reporting errors, near misses and incidents (score 1–5)	NSS
***Workplace pressure***		
Work pressure: Medical staff	Workplace pressure felt by medical staff (1–5; 5 is highest pressure)	NSS
Work pressure: Nursing staff	Workplace pressure felt by nursing staff (1–5)	NSS

PRR is the positive response rate; the % of respondents who answered agree orstrongly agree.

^a^The data for 2012–2013 and 2013–2014 were not available for this variable so it was estimated by mean imputation.

Data on *Ideas*, *Flexible*, *Job Design* and *Team Quality* were not available for the last two years of our NSS data window (2012/13 and 2013/14) due to questions being dropped or changed, or a change in the response format. We replaced the missing data with values calculated from the simple mean imputation technique (i.e. the mean of previous years’ values). The relatively small number of non-respondents and the high consistency of scores on the NSS management variables over time suggest that our estimates are unlikely to suffer from material non-response bias.

**Figure 2.**
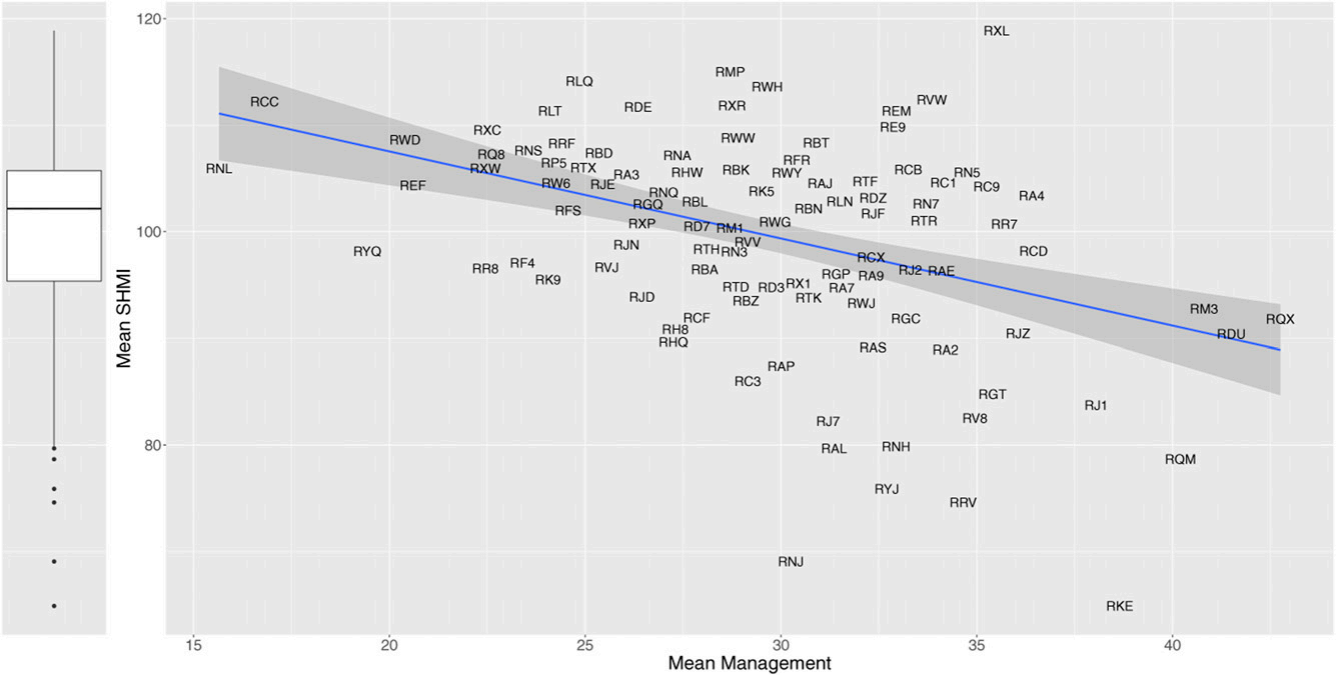
Mean relative mortality (SHMI) over the period 2010/11 to 2014/15 for NHS trusts. The vertical boxplot show high variation across NHS trusts. The scatterplot shows the relationship between trusts’ mean SHMI (relative patient mortality ratio) and their mean composite management practices score. The data points are represented by the NHS three-digit alphanumeric trust codes. The diagonal line is the regression fit, with its 95*%* confidence interval shown with grey shading.

Our analysis mainly uses individual practices and interactions between them. We also construct a composite management score for each trust by simply taking the mean of the four individual practices we identified above as of principal interest. For robustness, we also constructed a composite management score using principal component analysis, where the first component explains 74*%* of the variance. The correlation between the two measures is 0.99. The simpler composite of mean (average) management score is used for the X axis in Figure 2. We observe that higher composite management scores are related to lower levels of relative mortality. The fitted regression line shows that a unit increase on management (one percentage point increase in NSS respondents answering positively on each of the four variables, or on average across all) is associated with a reduction in SHMI of nearly 0.82 percentage points.

While our full dataset covers all hospital trusts, here we exclude specialist trusts, for example, tertiary cancer-treatment trusts, since their structures and workloads differ. To further control for structural characteristics, we include variables on Teaching Hospital status, Foundation Trust (FT) status and trust size (measured by the number of beds). FTs can exercise greater financial and managerial autonomy from direct government control. They are allowed to keep surpluses, which they can use to increase staff salaries and invest in improved patient services. Teaching Hospitals might incur higher costs and treat more complex or severe patients. They might also cause delay in the treatment process as consultants spend more time to train medical students.^2^ We also control for daycase surgery rates since recent advances in surgical techniques and anaesthesia have reduced overnight stays and infection rates and led to higher quality of care.^42^ We add year dummy (0, 1) variables to capture year-specific effects common to all trusts that may affect patient mortality outcomes.

We control for trust workforce skill-mix by including variables capturing the proportions of medical, nursing and support staff in a hospital’s workforce. Higher supply of skills can enable greater specialisation, better division of labour, timely scheduling of operation and more attention to patients, especially major and frail cases. All these might reduce mortality.^43^ We also control for workplace staff pressures, as perceived by doctors and nurses, since heavy clinical workload may create an environment where unsafe practices can occur, increasing hospital risk.^44^ We do not include the proportion of managers as a control variable. Across our years of interest, NHS Workforce statistics identify ‘managers’ as less than 3*%* of staff, which is small, and variation in the proportion of managers among trusts is also considered too low to be useful.^25^

Finally, it may be that better-managed trusts have a better culture of incident reporting. To minimise confounding bias, in our exploratory trees, we control for fairness and effectiveness of procedures for reporting errors, near misses and incidents at each NHS trust. Table 1 provides definitions and the data sources for all the variables and Table 2 gives their summary statistics.

**Table 2.** Summary statistics.

*Data/variable*	*N*	*Mean*	*St. Dev.*	*Min*	*25th percentile*	*75th percentile*	*Max*
SHMI	702	100.1	9.7	53.9	94.5	106.5	124.8
Teaching	974	0.188					
Foundation trust	974	0.555					
Daycase rate	974	76.807	10.411	15.079	74.624	82.807	99.083
Beds	970	696.975	380.551	7.000	435.000	904.000	2196.0
Medical staff (%)	972	11.379	3.711	4.674	9.717	12.526	100.000
Nursing staff (%)	973	31.809	4.337	12.040	29.235	34.079	50.071
Support staff (%)	974	28.954	8.203	9.986	26.667	31.592	244.400
Management: Decisions	818	26.522	5.933	8.000	22.000	30.000	54.000
Management: Feedback	818	29.144	5.585	7.000	25.000	33.000	50.000
Management: Ideas	817	37.378	7.034	16.000	32.000	42.000	63.000
Management: Communication	818	27.576	7.009	8.000	23.000	32.000	53.000
Flexible	817	66.350	5.500	47.000	62.000	71.000	82.000
Job design	817	3.393	0.071	3.170	3.340	3.440	3.600
Team quality	817	74.950	2.682	65.500	73.500	76.500	84.500
Appraisal	818	32.240	6.633	12.000	27.250	37.000	52.000
Supervisor	818	3.603	0.092	3.260	3.540	3.660	3.880
Work pressure (medical)	980	3.114	0.203	2.480	2.990	3.240	3.790
Work pressure (nurse)	987	3.199	0.166	2.480	3.110	3.310	3.620
Incident	641	3.490	0.092	3.170	3.430	3.550	3.770

The means of the Teaching and Foundation Trust variables represent the proportions of trusts that are of these types.

### Panel regression trees

Our primary goal in this paper is to explore potential management practice drivers of SHMI and their interactions. For example, we would like to understand whether the SHMI in trusts with comparatively higher scores on team quality *and* greater workplace flexibility is systematically different from that in trusts with either lower team quality *or* lower workplace flexibility. Traditional parametric methods such as panel regression models do not always offer straightforward interpretation of such intricate interplay of variables. We, therefore, resort to a class of non-parametric techniques, commonly known in machine learning literature as regression trees, that serve the purpose well. The tree mechanism involves recursively partitioning the predictor space into a pair of sub-regions based on simple rules, and then using the mean or median of the realised values (here SHMI) of observations (here trust-year datapoints) belonging to a region as the predicted value for a new observation that falls in that particular region. Usefully, the splitting decision rules, order of importance of selected predictors and their interactions are summarised in a visually intuitive and attractive way. The higher a variable appears in a tree, and/or the more times the algorithm selects it as the splitting variable, the greater its predictive importance.

Several tree growing algorithms are proposed in the statistics and machine learning literature. The most widely used are ‘CART’,^45^ which functions by maximising a statistical criterion over all possible predictors and split points simultaneously. These methods are often criticised for biased selection of variables which have many possible splits and missing values.^46^ Hothorn et al. (2006)^46^ propose a conditional inference framework that rectifies the problem of selection bias by choosing predictors for splitting based on a series of tests identifying statistically significant association between the responses (dependent variables) and predictors (independent variables). Partitioning (splitting) continues until there is no further statistically significant association between any of the predictors and the response variable. Over the recent decade, researchers have extended the unconditional inference framework to panel (longitudinal) data, where there are multiple observations over the same unit (here, a hospital trust) over years. Here, we use the Unbiased RE-EM Tree algorithm recently developed in Fu and Simono (2015),^47^ which is unbiased in nominating predictors for splitting. The Unbiased RE-EM Tree models also allow for variations among trusts due to unobserved trust attributes. We will train a panel tree model to identify (explore) optimal alignments of management practices that predict lower relative mortality rates. We will next test the statistical significance of the alignments using econometric panel regression methods.

## Results

### Exploring management practices

The regression tree technique arbitrarily selects from among any highly correlated variables to build a model that best predicts the outcome variable. In using the technique as an exploratory tool, one is required to classify the variables *in advance* into groups of relatively uncorrelated variables and apply the method to each group to better understand the data. Therefore, we a priori select from among the correlated variables to build a model and further examine the effect of replacing the explanatory variables with excluded variables to assess the robustness of the results. Here, a pair of variables is considered highly correlated if their correlation exceeds the threshold 0.80. Among the management variables, the correlation between *Decisions* and *Communication* exceeds the threshold. Of this pair, we exclude *Communication*. This gives results in the following model:

Regression Tree Model: SHMI ∼ Decisions + Feedback + Ideas + Appraisal + Flexible + Incident + Job Design + Supervisor + Team Quality

In using the method, one also has to set the significance threshold for splitting the predictors. Since we employ the panel regression technique as an explanatory tool, and later test the statistical significance of the alignments using traditional panel econometric methods, we set the significance threshold at 10*%* rather than the standard 5*%*. More discussion of this approach is given in recent, longer papers.^28–30^

**Figure 3.**
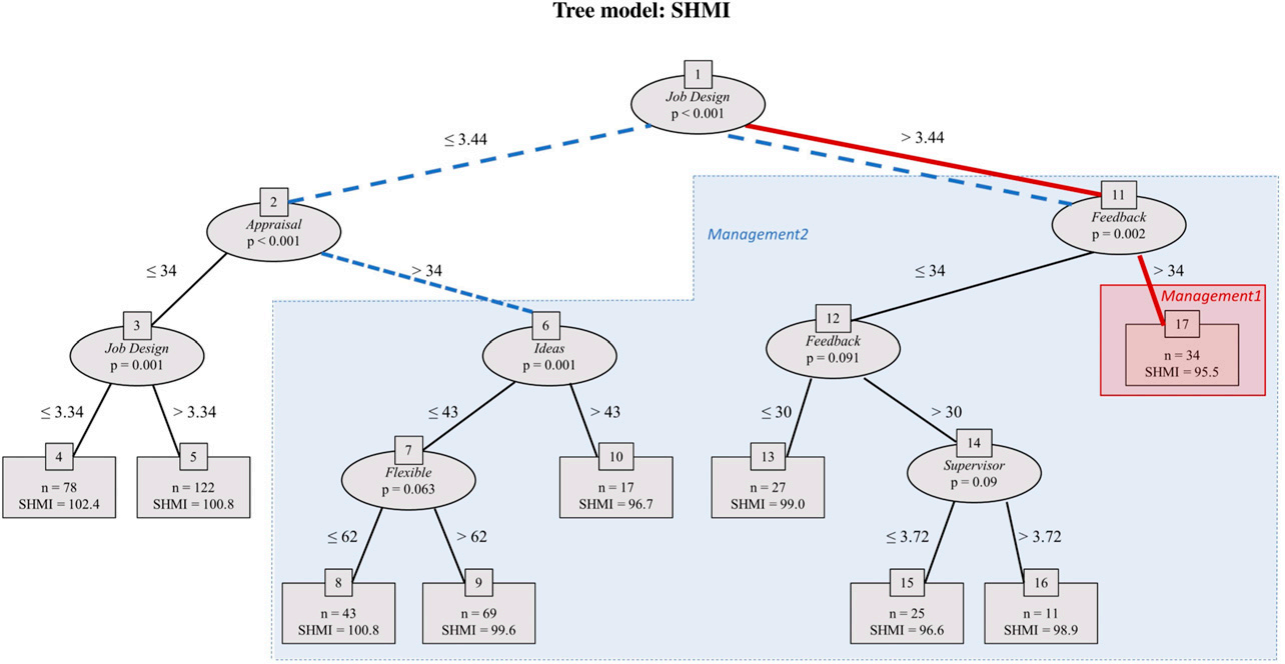
Tree model: SHMI. The unbiased regression tree includes all the variables listed in the regression tree model given in the text, with the dependent variable being SHMI. The maximum depth of the tree is set at four layers for simplicity. The higher a variable appears in the tree, the more predictively significant the variable is. *Job Design* appears at the initial node of the tree as the most predictively significant variable. *Appraisal* and *Feedback* appear in the next layer of the tree as the second most predictively significant variables. Variables missing from the tree, such as Management of *Decisions*, *Team Quality* and *Incident* reporting, are not predictively significant. Overall, the reasonably rich structure of the tree indicates the significance of management practices in predicting patient mortality. The two subsets of the tree picked out for testing in the multiple regression models (Table 3) are indicated with red/solid lines and blue/dashed lines.

Applying the unbiased RE-EM Tree with this significance threshold to the model above yields the tree shown in Figure 3. The tree has a rich structure: management variables predict a trust’s SHMI. *Job Design* appears in the initial node of the regression tree, suggesting that this variable is the most important predictor of SHMI. Trusts whose score for *Job Design* exceeds 3.44 have a lower SHMI (i.e. better performance): mean 97.9, versus 100.5 for those that do not. *Feedback* and *Appraisal* appear in the second layer, so are the second most predictively significant variables.

The tree reveals several alignments of management practices with low (i.e. good) mean SHMI. The lowest mean SHMI, 95.5, occurs in trusts in terminal node 17, characterised by the path between nodes {1, 11, 17}. This is highlighted on the tree with red solid lines and red shading. Here *Job Design* exceeds 3.44 and *Feedback* exceeds 34. Data points without these levels of management practices (i.e. everywhere else on the tree) have mean SHMI 100.3, so these management practices seem to be associated with around 5 percentage point lower relative deaths.

Terminal nodes 4 and 5 contain a set of datapoints with very poor (high) SHMI (mean 101.4, versus 98.6 for elsewhere on the tree). Here trusts have *Job Design* below 3.44 and *Appraisal* below 34. That cluster contrasts with the rest of the tree, highlighted with blue dashed lines and blue shading; here *Job Design* exceeds 3.44 or *Appraisal* exceeds 34.

As per the definitions in Table 1, these patterns suggest that trusts that receive high scores from staff on *Job Design* (the senior management set clear goals for employees, provide clear feedback on performance, and give staff an opportunity to participate in decision making) and *Feedback* (senior managers act on staff feedback) perform better on SHMI (i.e. have lower levels of relative mortality). In contrast, in trusts where such practices are poor, well-structured *Appraisal* reviews are absent, and performance on relative mortality is likely to be compromised.

It is important to note that it is not *Feedback* or *Appraisal* individually that matters, but it is their interaction with other variables such as *Job Design* that are associated with high or low levels of patient mortality. These are valuable pieces of evidence that are often missed by using conventional statistical techniques that fit a *global* model – applying a single predictive formula over the entire data space. This would be the case with going straight to the use of linear panel regression.

### Pattern testing

The explanatory panel regression tree (Figure 3) reveals several clusters (combinations of practices and their threshold levels) of management practices that might potentially drive SHMI. The final stage of our analysis is to test the statistical significance of the patterns of management practices suggested by the tree analysis, using econometric techniques. We include control variables for trust characteristics and estimate a series of models of the form:

**Panel Regression Model:** SHMI ∼ FT + Teaching + Daycase Rate + Beds + Medical Staff (%) + Nursing Staff (%) + Support Staff (%) + Work Pressure (Nurse) + Work Pressure (Medical) + Year dummies + Management

where *Management* is an indicator variable for the presence or absence of a set of particular practices at certain threshold levels as explained below; this is a dummy (0, 1) variable. As a step to deal with contemporaneous causality, the skill-mix variables, measures of staff pressures and management indices are all lagged by 1 year. The econometric literature favours fixed effects (FE) panel regression estimation to deal with unobserved heterogeneity in the units (here trusts). However, the FE estimator requires considerable ‘within-unit’ variation over time. Without this, the estimator is likely to generate large standard errors and suppress the explanatory power of ‘slow-moving’ variables of interest. The method would also force out time-invariant variables such as Foundation Trust or Teaching status.^48^ Our time window is fairly short and our explanatory variables are persistent over time, and we are interested in understanding possible impacts of the time-invariant variables. While emphasising the limits of the analysis, therefore, we use the random effects estimators.

Table 3 reports the regression model results. Column 1 includes only the control variables (i.e. everything except for the *Management* dummy): capturing trust characteristics, human capital, two measures of staff pressure, daycase rate and year dummies. The coefficient of *Teaching* status, − 8.698, is significant at 1%. Teaching trusts are associated with a SHMI 9 units (here 9 percentage points) lower than non-teaching trusts. Contrary to the conjecture stated earlier, teaching trusts may exercise extra care to avoid errors, have higher-level expertise available or be at the forefront of using new technologies and procedures, any of which reduces relative mortality. *Daycase Rate*, − 0.278, and *Medical Staff (%)*, −0.443, are both significant, at 1% and 5%, respectively, so are related to reduced mortality. In contrast, *Support Staff (%)*, 0.297 and *Work Pressure (nurse)*, 6.664 are positive and significant at 1*%*, tending to increase mortality. Support staff may lack adequate experience and incentives. Workload pressure may lead to poorer decisions, lower the quality of services and give rise to higher staff turnover rates. All these can compromise patient outcomes.^9^

**Table 3.** Random effects models: independent (outcome) variable is SHMI.

	(1)	(2)	(3)
Intercept	98.16 (16.93)^***^	104.3 (15.62)^***^	103.1 (16.03)^***^
FT	1.811 (1.104)	1.988 (1.060)^*^	2.080 (1.086)^*^
Teaching	−8.698 (1.875)^***^	−7.759 (1.881)^***^	−8.085 (1.887)^***^
Daycase rate	−0.278 (0.110)^**^	−0.255 (0.106)^**^	−0.242 (0.107)^**^
Beds	0.001 (0.002)	−0.000 (0.002)	0.000 (0.002)
Medical staff (%)	−0.443 (0.254)^*^	−0.348 (0.253)	−0.340 (0.252)
Nursing staff (%)	0.032 (0.143)	0.080 (0.142)	0.076 (0.144)
Support staff (%)	0.297 (0.137)^**^	0.288 (0.127)^**^	0.286 (0.129)^**^
Work pressure (nurse)	6.664 (2.512)^***^	4.306 (2.455)^*^	4.549 (2.473)^*^
Work pressure (medical)	−0.852 (1.669)	−1.228 (1.645)	−1.334 (1.681)
Management1		−10.73 (3.497)^***^	
Management2			−6.219 (2.711)^**^
*R*^2^	0.288	0.315	0.303
*Adj. R*^2^	0.281	0.307	0.295
*Num. obs.*	558	558	558

This table draws on the two regression tree to build several models of possible impacts of management practices on SHMI. Column 1 only includes a set of control variables capturing trust characteristics, human capital, two measures of staff pressure, daycase rate and year dummies. Column 2 adds an indicator (dummy) management variable, *Management1*, that captures the path {1, 11} in the first regression tree (Figure 3). The variable takes value one when the within-trust means of *Job Design* and *Feedback* exceed 3.44 and 34, respectively, and otherwise is zero. Column 3 instead adds *Management2*, a management indicator that captures all the paths in Figure 3 except the path {1, 2, 3}. The variable takes value one when the within-trust means of *Job Design* exceeds 3.44 or *Appraisal* exceeds 34, and otherwise is zero. The panel spans the years 2010/11 to 2014/15. The skill-mix variables and measures of staff pressures are all lagged by one year. Year dummies are included in all the regressions (but are not statistically significant and are omitted here for compactness). ^***^p < 0.01, ^**^p < 0.05, ^*^p < 0.1.

Column 2 adds a *Management* (dummy) variable, *Management1*, which picks out the first of the potential high-performance management practices part of the tree identified earlier (red/solid lines in Figure 3). To indicate membership of this cluster for regression analysis, we set *Management1* to the value one when the within-trust means of *Job Design* exceeds 3.44 and those of *Feedback* exceed 34, and otherwise to zero. In the regression results shown in Table 3, the coefficient estimate of *Management1* is −10.73, significant at 1*%*, that is, a reduction of around 11 percentage points in SHMI. This is double that estimated earlier just from the tree, that is, before potential confounds were included. In 2014–2015, NHS England reported 287, 000 deaths from 8, 700, 000 discharges from hospital (death is also a mode of ‘discharge’).^49^ The higher-performing cases picked out make up only 8*%* of the cases. If the other 92*%* performed at the same SHMI level as the cluster identified, the model would suggest nearly 13, 000 fewer (premature) deaths in a year. The evidence suggests when the clarity of tasks and goals are relatively high, tasks are allocated effectively and staff feedback are valued, relative mortality is indeed considerably and statistically significantly lower, even when considering the potential confounds.

Column 3 instead adds the *Management2* indicator variable that corresponds to the second potential high-performance part of the tree identified earlier (blue/dashed lines). Thus, *Management2* is one when *Job Design* exceeds 3.44 or *Appraisal* exceeds 34, and otherwise is zero. (Logically, this is the same as setting *Management2* to be zero when *Job Design* is less than 3.44 and *Appraisal* is less than 34, and otherwise to be one.) The estimate of the coefficient of *Management2* is −6.219, significant at 5*%*, so again mortality is indeed considerably lower in this cluster, after controlling for the potential confounding variables. Our results support that clusters of management practices may matter for relative mortality.

## Discussion

Our results are consistent with a growing body of empirical and experimental evidence from a range of settings that ‘management practices matter for performance’.^22,28,29,32^ Increasing evidence suggests improvement in management practices can save lives. They contrast with the recent null finding from investigating the relationship between SHMI and management quality measures by a simple aggregate of NSS scores.^25^

The results in the previous section provide evidence in favour of the hypothesis that clusters of management practices may matter for relative mortality. Our evidence suggests any effort to improve patient mortality might be supported by adopting a holistic portfolio of well-aligned management practices, particularly those practices relating to the design and clarity of tasks, task allocation, continuous reviewing of practices, workplace flexibility and engagement of staff in important decisions. In particular, the variables appearing high in the tree (Figure 3), so with high predictive power, are those that are intuitively most relevant for patient mortality such as *Job Design*, *Appraisal* and *Feedback*.

Strengths of our analysis include the construction of a five-year time-series dataset covering the population of public hospital providers, an opportunity afforded by the working with NHS sources. The NHS staff survey (NSS), open to all staff across all trusts, is also a strength. This large and rich dataset has allowed us to explore multiple parameters across a whole acute-hospital healthcare system. A further strength is our use of recent developments in machine learning to suggest specifications for econometric dummy-variable panel regression analyses, enabling us to pick out interacting clusters of variables and the nonlinear impact of threshold levels of these.

There are several caveats to our study. A limitation relates to the measurement of the management practices. While the NHS staff survey, on average, has a response rate of around 50*%* of staff to whom it is sent,^8^ there can be legitimate questions on how adequately the responses capture the quality of practices in the trusts. A second limitation is that our panel is fairly short and most variables in the sample are persistent over time. With a longer sample, there would be more variation in the data and one could run fixed effects regressions to control for unobserved trust heterogeneity. Finally, we cannot make claims for causality from our study; the evidence is only indicative. Good management practices appear to be conducive to lower patient mortality. Causality might be examined through randomised controlled or natural experiments or through using valid instrumental variables. The COVID-19 shock could serve as a natural experiment. An implication of our analysis is that trusts that are members of different clusters of management practices might be expected to perform differently in response to the shock. Specifically, trusts that scored higher on those management practices associated with higher performance prior to the pandemic might be expected, *ceteris paribus*, to respond better to demand pressures and have lower relative mortality during the pandemic. We aim to pursue this line of research in the near future.

Our analysis builds on the well-documented heterogeneity in management practices across hospitals. This raises a deeper question about *why* management practices differ to such an extent across hospital organisations. This question shapes our ongoing research. We also aim to further our understanding of complementarities between management practices and other organisational routines, which may affect relative patient mortality.

### Relevance of the results

Our analysis offers evidence on the association, and potential impact, of management practices on relative patient mortality. In the United Kingdom, high-profile failures in healthcare have led to a series of government-sponsored investigation over the last 50 years.^50^ In particular, in the last decade, the results were published from two very substantial and influential official investigations into poor patient safety and mortality in the English NHS: The Francis Report and the Keogh Review.^51,52^ ^1^

Similar investigations into major patient safety failures have been conducted in other developed countries including the United States, Canada, Australia and New Zealand. A common theme when looking across these was lack of or poor management systems for quality appraisal, incident reporting and performance management: problems were often longstanding, and often known, but were not addressed – the feedback and learning practices were poor.^53^ Other countries have conducted enquiries into specific patient safety issues (e.g. in France into infected blood and defective breast implants. In Ireland, the government has used risk-adjusted mortality rates (similar to SHMI) to highlight a hospital organisation with consistently poor relative mortality, although they did not seek a connection with management practices.^54^

Figure 3 picks out the clusters of management practices (labelled as *Management1* and *Management2*) which our NHS data show to be important (and which were confirmed as statistically and materially significant by the regressions in Table 3). These suggest the narrative: where *Job Design* is good, performance is good and it is even better if *Feedback* from staff is acted on; where *Job Design* is not good, then performance is still good if the *Appraisal* process is well structured and is better still if *Ideas* from staff for improving services are encouraged.

We suggest these results are in tune with the recommendations of Francis and Keogh and give some statistical weight and generalisability to their qualitative findings from a small number of trusts. Francis noted a strong culture of senior management ignoring or failing to act on signals from staff (and patients and other parts of the health system) – including whistleblowers – with senior management claiming that staff were not speaking up to draw their attention to problems. One of the Report’s many recommendations was that the clinical staff appraisal process should require evidence of commitment to patient care, and feedback processes should be widely strengthened. Prominent amongst the themes in the Keogh analysis were the importance of (i) genuinely listening to views of staff (and patients) and (ii) engaging staff in how to improve delivery of services and tapping into their natural innovation and energy.

Although Francis and Keogh advise against trying to extrapolate from statistical analysis to numbers of avoidable deaths,^51,52^ in this paper we made a quick calculation in order to help readers interpret the potential effect sizes of patterns we found to be statistically significant. Our estimate was 13,000 avoidable deaths per year, if all trusts performed at the level of those in the *Management1* cluster *and our results reflected causality*. We suggest the potential effect is large enough to justify further research.

## Note

1. The Francis Report followed the government-commissioned public inquiry (the Francis Inquiry) into scandalously poor care and high mortality rates at the Mid-Staffordshire Hospital Trust. The scandal contributed to the resignation of the NHS Chief Executive and prompted the Prime Minister to order the Keogh Review into hospital mortality rates, which sent teams of investigators into fourteen acute trusts; their performance on SHMI was an important factor in selection. As a result, eleven of these trusts were put under ’special measures’ for remedial action. These two reports were not aiming for statistical significance, but looking for common themes within the wide range of local contextual factors.
